# Supplementary material for: The interactome of intact mitochondria by cross-linking mass spectrometry provides evidence for coexisting respiratory supercomplexes
Source: Mol Cell Proteomics. 2017 Dec 8;17(2):216–32. doi: 10.1074/mcp.RA117.000470 (PMC5795388; doi:10.1074/mcp.RA117.000470)
Supplement: Supplemental Data [file supp_RA117.000470_133922_0_supp_23978_fzffwf.zip › spectra_annotation/mito_spectra_annotation/239-1-1-1-4-1.pdf]

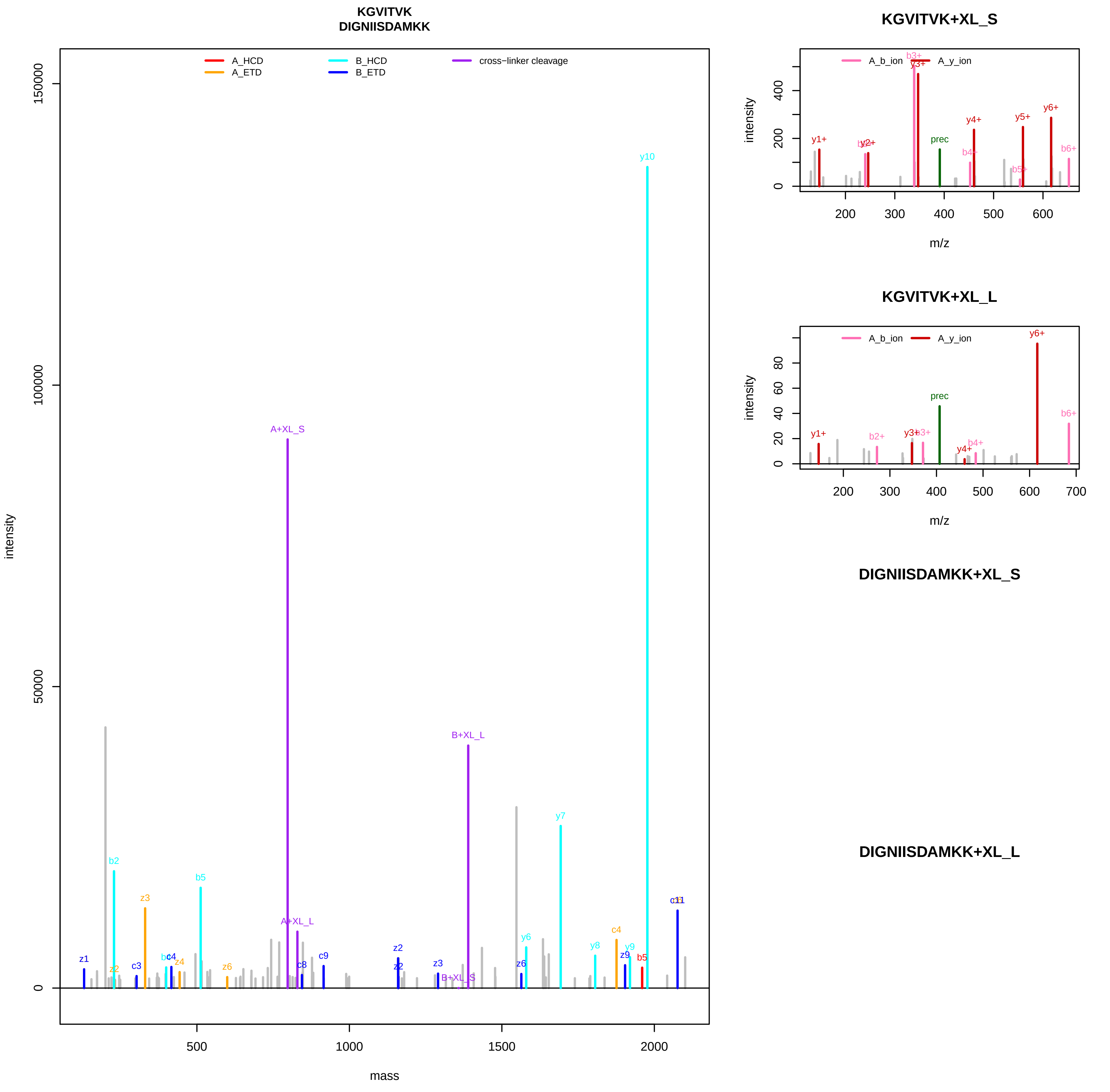
KGVITVK
DIGNIISDAMKK
A_HCD
A_ETD
B_HCD
B_ETD
cross−linker cleavage
intensity
mass
0
50000
100000
150000
500
1000
1500
2000
y10
A+XL_S
B+XL_L
A+XL_L
B+XL_S
y7
b2
b5
z3
y6
y8
y9
c4
z9
b5
c11
z1
z2
c3
z4
z6
c8
c9
z2
z3
z6
KGVITVK+XL_S
KGVITVK+XL_L
DIGNIISDAMKK+XL_S
DIGNIISDAMKK+XL_L
A_b_ion
A_y_ion
m/z
prec
y1+
y2+
y3+
y4+
y5+
y6+
b2+
b3+
b4+
b5+
b6+
